# Prevalence and risk factors of anemia in children aged from 6 to 59 months in Togo: analysis from Togo demographic and health survey data, 2013–2014

**DOI:** 10.1186/s12889-019-6547-1

**Published:** 2019-02-20

**Authors:** Aboubakari Nambiema, Alexie Robert, Issifou Yaya

**Affiliations:** 1 Centre Africain de Recherche en Epidémiologie et en Santé Publique (CARESP), Lomé, Togo; 2 Aix-Marseille Université, Marseille, France

**Keywords:** Anemia, Prevalence, Risk factors, Children, Togo

## Abstract

**Background:**

Anemia in children continues to be a major public health challenge in developing countries and particularly in Sub-Saharan Africa. Anemia has serious consequences on the growth and development of the children in the early stages of life. This study aimed to determine the prevalence and associated factors of anemia among children from 6 to 59 months in Togo.

**Methods:**

Data from the Togo Demographic and Health Survey 2013–2014 were used for this study. This nationally representative survey provided data on a wide range of indicators such as mother and child health, nutrition and other characteristics. Anemia status was determined using hemoglobin level (Hb < 11.0 g/dl), and the weighted prevalence of childhood anemia along with 95% confidence intervals were provided. Data were analyzed using logistic regression models to estimate odds ratios (OR) and their 95% confidence intervals (95% CI) for associated factors.

**Results:**

Two thousand eight hundred ninety children aged 6–59 months were included in this analysis. The weighted prevalence of anemia was 70.9% [95% CI = 68.8–73.1] with 2.6% [95% CI = 2.0–3.3] of severe anemia among these children. In the multivariate analysis, the adjusted odds ratio (aOR) for anemia was 0.33 [95% CI = 0.26–0.42] in children aged from 24 to 42 months and 0.22 [95% CI = 0.17–0.29] in children aged from 43 to 59 months. Children’s malaria status was strongly associated to childhood anemia with an aOR of 3.03 [95% CI = 2.49–3.68]. The secondary level of education and more for the mother was associated to childhood anemia with an aOR of 0.67 [95% CI = 0.52–0.86]. The aOR for children whose mother had anemia was 1.62 [95% CI = 1.30–2.02].

**Conclusion:**

This study has highlighted the high prevalence of childhood anemia in Togo and revealed that younger children and maternal anemia were positively associated to childhood anemia whereas age of children and high level of maternal education were negatively associated to childhood anemia.

## Background

According to the World Health Organization (WHO), anemia is one of the ten most serious health problems in the world [1]. Frequently observed among children aged 6 to 59 months and in pregnant women who are the most vulnerable group, anemia is defined as a hemoglobin level below 11.0 g/dl [1, 2]. It corresponds to a state in which the number of red blood cells is low, or their ability to carry oxygen (i.e. hemoglobin) is so poor, in order to satisfy the physiological needs of the organism. In 2011, the WHO [2] estimated that about 273.2 million children aged from 6 to 59 months in the world were suffering from anemia, with an overall prevalence of 42.6%. However, this disproportionately affects the regions of the world. Sheltering approximately 84.5 million 6–59 months aged children suffering from anemia, Sub-Saharan Africa remains the most affected region with a prevalence reaching 62.3%.

The early treatment of anemia and its eradication are a public health goal as well as a major school challenge because it could not only improve growth, but also the intellectual capacities of children [3]. Indeed, the consequences of the anemia among preschoolers are serious [4–7] and include: impairment of cognitive function, impaired motor development and growth, declining academic performance, decreased immune function which exposes children to infections, decreased responsiveness and activity, and fatigue. These can irreversibly compromise the future development of a child. This situation has awoken a particular interest both nationally and internationally, leading to the implementation of prevention programs through food fortification and intermittent iron supplementation [8, 9].

However, the success of these community programs in Sub-Saharan Africa require a good knowledge of the risk factors of anemia. Several studies have been conducted in this area [10–13] and showed that anemia is present in 60.2 to 87.8% of children from 6 to 59 months. They highlighted a link between anemia among preschoolers and several factors including individual (age, childhood malaria, female sex), maternal (educational level) and contextual characteristics such as household income, living environment (urban/rural). A study based on data from Demographic and Health Survey (DHS) of 11 African french-speaking countries [10] showed that protein-energy malnutrition strongly contributed to the occurrence of anemia among children. In another study conducted in Ghana, the prevalence of anemia was high among preschool children aged less than 2 years [11]. In a recent study of anemia risk factors in children aged 6–59 months in sub-Saharan Africa, Moschovis et al. [14] found associations between child anemia and individual (age, female sex, birth order), maternal (maternal anemia, mother’s age mother’s body mass index) and contextual (household income, family structure) characteristics of the child.

In Togo, the 2013–2014 DHS revealed that the prevalence of anemia among children aged from 6 to 59 months was also high and anemia affected about 70.0% of these children, of which 2.0% were suffering from a severe form [15]. With a prevalence higher than 40.0%, WHO considers anemia in children 6–59 months as a severe public health problem [1, 16]. Furthermore, Togo is part of the African region where the prevalence of anemia among children aged 6 to 59 months has been estimated at over 72% [10]. Nevertheless, apart from some studies conducted in hospital settings [17, 18], the topic of anemia in children aged 6–59 months is mostly unexplored at the national level in Togo.

This study aimed to determine the prevalence and associated factors of anemia among children aged from 6 to 59 months in Togo. This study on the risk factors of anemia in children aged from 6 to 59 months is the first of its kind in Togo, and can provide reliable information to help develop new strategies for the prevention of anemia among the children in this country.

## Methods

The data used in this study is extracted from the third Togo Demographic and Health Survey (DHS-III) conducted in 2013–2014. DHS-III is part of the DHS program, led by the United States Agency for International Development (USAID), which has been conducting household surveys in developing countries since 1984 [19]. Details about the survey can be found in the 2013–2014 DHS report [15]. DHS are regularly conducted among large samples of households’ representative of the population of each country. Their goal is to provide governments with recent and reliable information on population health indicators in order to allow for adaptation of health policies. One of the major interests of these surveys is the homogeneity of their methodology in different countries, which permits in most cases, to compare situations and obtain a global vision [10]. The DHS deals with many health issues, including reproduction, child health (breastfeeding and nutrition, vaccinations, anemia, acute respiratory infections and diarrhea), malaria and the Human Immunodeficiency Virus (HIV) [15].

According to the Report of the DHS 2013–2014 in Togo [15], the DHS-III aims to produce results representative of the whole country, of separate urban and rural environments, from the city of Lomé (the capital city of Togo), and from each of the five regions of Togo.

As anemia has a high prevalence in children under 5 years old, and causes serious impairment to the development of preschoolers, we limited our study to the age group of 6–59 months old. In the Togo DHS 2013–2014, 3378 children between 6 and 59 months were evaluated. Of these, 2890 children had anemia test results, and were included in the study”. Survey weights were used in analyses in order to account for the unequal probability of selection of households induced by the sampling scheme and non-response. A detailed explanation of the weighting procedure can be found in the Demographic and Health Survey Methodology report [20].

### Outcome variable

During the DHS-III, anemia was tested by performing blood samples in every second household in the study population. The tests were performed using a portable hemoglobin meter (HemoCue) device which, in less than a minute, could give the hemoglobin level in grams per deciliter of blood, and finally allow to record the value in the questionnaire [10].

According to WHO, in children aged from 6 months to 5 years old, a hemoglobin concentration level below 11.0 g/dl corresponds to anemia, a level below 7.0 g/dl is considered as severe anemia, a level between 7.0 g/dl and 9.9 g/dl is considered as moderate anemia and a level between 10.0 g/dl and 10.9 g/dl is considered as mild anemia [2]. Thus, the dependent variable was coded into two levels. A child was classified as anemic if his hemoglobin level was below the WHO threshold of 11.0 g/dl.

### Independent variables

#### Variables related to child’s characteristics

Among the selected independent variables, some were related to the child such as sex, age (divided into three categories: 6–23 months, 24–42 months and 43–59 months), taking iron supplements, nutritional status and malaria diagnosis.

Nutritional status was estimated from anthropometric measures of height and weight of the child. For each individual, his height / weight ratio was compared to the median for the population. From this, nutritional status was divided into three categories. A detailed explanation of the procedure can be found in the Reference Guide on Nutrition Indicators for Development [21]. Thus, if an individual had a standard deviation lower than − 2, he was considered as malnourished; if the standard deviation was between − 2 and 2, he was considered as normal and if the standard deviation was greater than 2, he was considered as overweight.

Malaria diagnosis was made using the rapid diagnostic test which gave a result either positive meaning that the child had malaria or negative if the child did not have malaria [10].

#### Variables related to the child’s mother

Variables relating to the child’s mother were also selected: anemia (anemic or not), educational level (no education, primary or secondary school level and above) and economic activity (currently working or have worked in the last 12 months, not working).

#### Variables related to household characteristics

The child’s household income, region of residence, place of residence (urban/rural) and whether or not his father was alive were included in the statistical analysis.

### Statistical analysis

We conducted descriptive data analysis in order to describe the main characteristics of our sub-sample of young Togolese children suffering from anemia. In these analyses, we performed Chi-Square test of Rao-Scott to determine whether there was a significant difference between observed and expected frequencies.

To evaluate the effect of each of individual, maternal and contextual characteristics on anemia in Togolese children aged from 6 to 59 months old, logistic regression models were used to estimate odds ratios (ORs) and their 95% confidence intervals (95% CI).

The association between the independent variables and anemia in the children were first examined using bivariate logistic regression. All significant variables with *p*-value of Wald test less than 0.20 were included in the multivariate model. The non-significant variables in the bivariate analysis whose associations with anemia had been shown in the literature were also introduced into the multivariate regression models. The final model was obtained using a manual variable selection method based on the *p*-value in order to obtain the parsimonious model.

All analyses were performed using SAS (Statistical Analysis Software) version 9.4.

## Results

### Characteristics of study population

Two thousand eight hundred and ninety (2890) children aged 6–59 months were included in this analysis. Children aged 6–23 months were the most represented (36.3%). The proportion of boys and girls in the sample was similar with respectively 1456 (50.4%) and 1434 (49.6%). Two thousand and eighty-five (2085) children (72.6%) had not taken iron supplements and 213 (7.4%) of them were malnourished. During the survey more than one-third of the children, 1067 (37.0%) were tested positive for malaria.

Overall, 1522 children (52.7%) lived in poor household and 2111 children (73.0%) in the rural areas. Almost a quarter of the children, 701 (24.3%), in the sample were from the Savanes region. Most children’s, 2824 (97.8%) fathers were alive during the survey.

Concerning the characteristics of the children’s mothers, 1345 mothers (46.5%) had no education whereas 556 (19.2%) attended secondary school or more. Most of mothers (85.5%) were employed and 1232 mothers (42.8%) had anemia (Table 1).

**Table 1:** Prevalence of anemia (%) among Togolese children aged 6–59 months by demographic and household characteristics

Factors	n (%)	Anemia (n)	Unweighted Prevalence of anemia [95% CI]	Weighted Prevalence of anemia [95% CI]	*p*-value
Total	2890 (100.0)	2052	71.0 [69.0–73.0]	70.9 [68.8–73.1]	
Child related factors					
Sex of children					0.2863
Male	1456 (50.4)	1049	72.0 [69.7–74.7]	71.9 [69.1–74.8]	
Female	1434 (49.6)	1003	69.9 [67.3–72.6]	69.9 [67.1–72.7]	
Age of children					< 0.0001
6–23 months	1048 (36.3)	887	84.6 [82.4–86.9]	84.0 [81.4–86.5]	
24–42 months	987 (34.1)	660	66.9 [63.9–69.8]	66.4 [63.4–69.5]	
43–59 months	855 (29.6)	505	59.1 [55.3–62.9]	59.7 [55.4–63.9]	
Nutritional status					0.0059
Malnourished	213 (7.4)	171	80.3 [74.9–85.7]	80.6 [74.8–86.3]	
Normal	2606 (90.2)	1826	70.1 [68.0–72.2]	70.0 [67.7–72.3]	
Overweight	71 (2.4)	55	77.5 [67.7–87.2]	77.4 [67.1–87.7]	
Iron supplements intake					0.4983
No	2085 (72.6)	1494	71.7 [69.4–73.9]	71.4 [68.9–73.9]	
Yes	788 (27.4)	547	69.4 [65.8–73.1]	69.8 [65.7–73.8]	
Children had malaria					< 0.0001
No	1820 (63.0)	1173	64.5 [62.0–66.9]	64.5 [61.9–67.2]	
Yes	1067 (37.0)	876	82.1 [79.7–84.5]	82.4 [79.9–84.9]	
Maternal factors					
Mother’s education status					< 0.0001
No education	1345 (46.5)	990	73.6 [70.8–76.4]	74.2 [71.1–77.2]	
Primary	989 (34.2)	710	71.8 [68.8–74.8]	72.7 [69.3–76.0]	
Secondary and more	556 (19.2)	352	63.3 [59.1–67.5]	61.6 [57.4–65.9]	
Mother’s occupation status					0.1650
Not working	419 (14.5)	309	73.7 [69.4–78.1]	73.9 69.3–78.5]	
Working	2470 (85.5)	1742	70.5 [68.4–72.7]	70.4 [68.1–72.7]	
Mother’s anemia status					< 0.0001
No	1649 (57.2)	1104	66.9 [64.3–69.6]	66.9 [63.9–69.9]	
Yes	1232 (42.8)	940	76.3 [73.8–78.8]	75.8 [72.8–78.7]	
Household factors					
Wealth index					< 0.0001
Poor	1522 (52.7)	1107	72.7 [70.0–75.5]	74.2 [71.4–77.1]	
Middle	528 (18.3)	397	75.2 [71.1–79.3]	74.6 [70.3–79.0]	
Rich	840 (29.1)	548	65.2 [61.5–68.9]	65.2 [61.4–69.0]	
Father’s alive					0.7447
No	64 (2.2)	45	70.3 [59.7–80.9]	69.0 [56.9–81.1]	
Yes	2824 (97.8)	2006	71.0 [69.0–73.0]	71.0 [68.8–73.2]	
Place of residence					0.0002
Rural	2111 (73.0)	1542	73.0 [70.7–75.4]	73.9 [71.4–76.4]	
Urban	779 (27.0)	510	65.5 [61.6–69.3]	65.3 [61.4–69.2]	
Region of residence					0.0259
Savanes	701 (24.2)	478	68.2 [63.6–72.8]	69.2 [64.4–74.0]	
Kara	434 (15.0)	336	77.4 [72.7–82.1]	77.0 [71.9–82.2]	
Centrale	412 (14.2)	306	74.3 [69.6–79.0]	74.5 [69.6–79.4]	
Plateaux	519 (18.0)	380	73.2 [68.9–77.5]	73.2 [69.3–77.2]	
Maritime (without Lomé)	334 (11.6)	234	70.1 [63.9–76.3]	69.8 [62.9–76.7]	
Lomé town	490 (17.0)	318	64.9 [60.2–69.6]	65.6 [61.1–70.0]	

*p*-value: the *p*-value of Chi-Square Rao-Scott test

#### Prevalence of anemia

The overall prevalence of anemia among children aged from 6 to 59 months was 70.9% [95% CI = 68.8–73.1] of which 25.6% [95% CI = 23.7–27.5] had mild anemia, 42.7% [95% CI = 40.4–45.0] had moderate anemia and 2.6% [95% CI = 2.0–3.3] had severe anemia. Table 1 provides the characteristics of children, their mother, their household, and the distribution of anemia across child related factors, maternal factors and household factors. A higher proportion of anemia was observed among children younger than 24 months (84.0% [95% CI = 81.4–86.5]; *p* < 0.0001), malnourished (80.6% [95% CI = 74.8–86.3]; *p* = 0.0059), and suffering from malaria (82.4% [95% CI = 79.9–84.9]; *p* < 0.0001). We also observed higher proportion of anemia in children whose mothers suffered from anemia (75.8% [95% CI = 72.8–78.7]; *p* < 0.0001) or had no education (74.2% [95% CI = 71.1–77.2]; *p* < 0.0001). In addition, this proportion was also significantly higher for children who lived in rural areas (73.9% [95% CI = 71.4–76.4]; *p* = 0.0002) or in the Kara region (77.0% [95% CI = 71.9–82.2]; *p* = 0.0259) (Table 1).

### Factors associated with anemia

Table 2 shows results of bivariate and multivariate analyses for assessing associations between childhood anemia and potential risk factors.

**Table 2:** Factors associated with anemia in children aged 6–59 months in Togo

Factors	Model 1		Model 2	
	cOR [95% CI]	pW	aOR [95% CI]	pW
Child related factors				
Sex of children		0.3281		
Male	1.00		–	
Female	0.91 [0.76–1.09]		–	
Age of children		< 0.0001		< 0.0001
6–23 months	1.00		1.00	
24–42 months	0.38 [0.30–0.47]		0.33 [0.26–0.42]	
43–59 months	0.29 [0.22–0.37]		0.22 [0.17–0.29]	
Nutritional status		0.0048		
Normal	1.00		–	
Malnourished	1.82 [1.23–2.70]		–	
Overweight	1.45 [0.80–2.64]		–	
Iron supplements intake		0.5753		
No	1.00		–	
Yes	0.94 [0.75–1.18]		–	
Children had malaria		< 0.0001		< 0.0001
No	1.00		1.00	
Yes	2.55 [2.12–3.07]		3.03 [2.49–3.68]	
Maternal factors				
Mother’s education status		< 0.0001		0.0027
No education	1.00		1.00	
Primary	0.94 [0.75–1.17]		1.01 [0.80–1.28]	
Secondary and more	0.56 [0.45–0.71]		0.67 [0.52–0.86]	
Mother’s occupation status		0.1749		
Not working	1.00		–	
Working	0.84 [0.66–1.08]		–	
Mother’s anemia status		< 0.0001		< 0.0001
No	1.00		1.00	
Yes	1.53 [1.24–1.89]		1.62 [1.30–2.02]	
Household factors				
Wealth index		0.0003		
Poor	1.00		–	
Middle	1.02 [0.79–1.32]		–	
Rich	0.65 [0.52–0.81]		–	
Father’s alive		0.6838		
No	1.00		–	
Yes	1.13 [0.63–2.00]		–	
Place of residence		0.0002		
Rural	1.00		–	
Urban	0.66 [0.53–0.82]		–	
Region of residence		0.0104		
Savanes	1.00		–	
Kara	1.31 [1.03–2.16]		–	
Centrale	1.49 [0.92–1.86]		–	
Plateaux	1.21 [0.89–1.64]		–	
Maritime (without Lomé)	1.03 [0.69–1.53]		–	
Lomé town	0.84 [0.62–1.13]		–	

pW: *p* value of Wald’s Chi-Square test cOR: crude OR aOR: adjusted OR 95% CI: 95% confidence interval

In bivariate analysis, we found that the following variables were associated with childhood anemia (Model 1): age of children, child nutritional status, child malaria status, mother’s education, mother’s anemia status, household wealth index, place of residence and region of residence. No association was apparent between childhood anemia and sex of children, taking iron supplements, mother’s occupation status or whether or not the child’s father was alive.

Model 2 in Table 2 shows the odds ratios from multivariate logistic regression for assessing associations between different factors and anemia among children aged 6–59 months in Togo. The child’s late age and his mother’s high-level education were negatively associated to childhood anemia whereas malaria in the child and anemia in his mother were positively associated to childhood anemia. The adjusted odds ratio (aOR) for anemia was 0.33 [95% CI = 0.26–0.42] in children aged from 24 to 42 months and 0.22 [95% CI = 0.17–0.29] in children aged from 43 to 59 months. The OR for anemia was three times higher in children with malaria compared to those without anemia (aOR = 3.03 [95% CI = 2.49–3.68]). The odds of childhood anemia among children whose mothers had anemia was higher compared to those whose mothers did not have anemia (aOR = 1.62 [95% CI = 1.30–2.02]). Concerning mother’s education, we observed that secondary level education or more was negatively associated with the childhood anemia (aOR = 0.67 [95% CI = 0.52–0.86]). The sex of children, their nutritional status, whether or not they took iron supplements and all household factors were not associated with childhood anemia in the multivariate model.

## Discussion

This study adds evidence to the literature concerning prevalence and risk factors of childhood anemia in Sub-Saharan Africa countries.

To the best of our knowledge, this is the first study in Togo estimating prevalence and risk factors of anemia in children aged from 6 to 59 months. The results of this study have shown a high prevalence of anemia among Togolese children aged from 6 to 59 months, making this a serious public health issue [1, 16]. In Togo, staple foods consist of corn, millet, cassava and rice which are rich in carbohydrates. This low diversification of food substitutes along with a low iron-rich diet could explain this high rate of anemia despite the high rates of breastfeeding. Moreover, there are rarely specific food formulas that account for the nutritional needs of the child. Previous studies have shown similar results ranging from 72.4% in 11 French-speaking African countries to 78.4% in Ghana [10, 18, 22].

When examining associations between risk factors and childhood anemia, we showed that anemia was associated with both characteristics of the child and those of his mother. The negative association between the age of the child and anemia has been showed by Ngesa and Mwambi who reported a lower risk of anemia in older children [23]. Magalhães and Clements [13] in a National cross-sectional household-based demographic health surveys in West Africa reported the same trend of anemia prevalence by age. This could be explained by the fact that children who are getting older receive a diet which is richer and complete, with a sufficient intake of iron which could prevent the occurrence of anemia in the child.

Concerning the child’s malaria status, those who had malaria were found to have higher risk of anemia as compared to their counterparts who did not have malaria. Increased hemolysis or decreased red blood cell production rates, could explain the higher risk of anemia among malaria patients compared to non-malaria patients [24]. Similar results have been reported in several other studies [23, 25, 26]. Regarding iron intake, our results did not show association between iron supplements intake and childhood anemia, unlike the literature. This may be due to the fact that children did not take iron supplements continuously or that the amount which was taken by the children was low. However, the quantity of iron was not also indicated because the DHS study did not provide information concerning iron supplementation. As we lacked this information on iron supplementation, it could be biased to conclude on the effectiveness of this strategy. Therefore, there is a need to rethink how to integrate iron supplementation of children under 5 into dietary habits. In addition to this, there is no specific public health policy in Togo to prevent anemia, however, in most of the time, iron supplementation is proposed as an adjuvant treatment during the therapeutic management of malaria in children.

Regarding the mother’s characteristics, we found that the mother’s education level was negatively associated with anemia in children. Indeed, children whose mothers had a secondary school education level or higher were less likely to be anemic. These results were in similar to the results of previous studies on the effect of maternal schooling on children’s anemia [23, 27]. Mothers with a high level of education are more likely to know about good dietary practices and take into account the nutritional values of foods. Moreover, they understand better and provide a healthy and hygienic diet for their children.

As for the relationship between anemia in the child and anemia in his mother, our study showed that, maternal anemia was consistently associated with the occurrence of child anemia. This finding can be explained by the fact that mothers and their children most often share a common environment, which could involve common exposures to some risk factors. Similar findings were observed in other studies [27, 28]. Furthermore, Al-Qaoud and colleagues [29] also showed this association among mothers of Kuwaiti preschool children.

The present study has some limitations that should be noted. The study design does not allow for cause-and-effect relationships to be established. In addition, we did not have data on child nutrition such as frequency of fruit, vegetable and meat consumption, which is important for a better understanding of the epidemiology of childhood anemia. The use of capillary blood in the place of venous samples may introduce a source of bias owing to the possibility of hemoglobin dilution with extracellular fluid through manipulation of the subject’s finger when the technician pricks the skin to collect the blood drop. Despite the limitations of this technique, it has great recognized practical advantages and does not compromise the quality of diagnosis at the population level [30, 31].

This study has some strengths. The results are generalizable to the whole country. Indeed, we used representative survey data based on a validated questionnaire and methodology [20]. Moreover, these results appear consistent with the literature and is the first study in Togo examined prevalence and risk factors of anemia in children from 6 to 59 months.

Since anemia in children under 5 is considered a public health problem and WHO recommends iron supplementation, particularly for children 6 to 23 months of age, we could make the following recommendations to Togolese government based on the results of this study: Government must strengthen iron supplementation interventions targeting young children and those suffering from malaria. Interventions are also recommended to encourage populations to diversify children’s foods or to enrich them with iron.

## Conclusion

This study has highlighted a high prevalence of childhood anemia in Togo. The findings showed regional differences in the prevalence of anemia among children, with the highest rate being observed in the North region of the country as in Kara region. The results from this study have shown that malaria in children and maternal anemia were positively associated to childhood anemia whereas age of children and high level of maternal education were negatively associated to childhood anemia. In terms of public health, the results of the present study could help to improve the targeting of interventions that integrate and take into account identified risk factors, particularly among children under five years of age.

## Abbreviations

95% CI: 95% confidence intervals
aOR: Adjusted odds ratio
cOR: Crude odds ratio
DHS: Demographic and Health Surveys
Hb: Hemoglobin
HIV: Human Immunodeficiency Virus
USAID: United States Agency for International Development
WHO: World Health Organization
